# Risk factors for abscess formation during or following endodontic treatment in an outpatient setting

**DOI:** 10.1038/s41598-026-59391-0

**Published:** 2026-06-30

**Authors:** Thomas Gerhard Wolf, Janika Wollmann, James Deschner

**Affiliations:** 1 Department of Periodontology and Operative Dentistry, University Medical Center of the Johannes Gutenberg-University Mainz, Augustusplatz 2, Mainz, 55131 Germany; 2 Department of Restorative, Preventive and Pediatric Dentistry, School of Dental Medicine, University of Bern, Bern, Switzerland

**Keywords:** Periapical periodontitis, Case-control studies, Dental abscess, Root canal therapy, Maxillofacial infections, Postoperative complications, Risk factors, Retrospective studies, Dental pulp, Outcomes research, Clinical trial design, Pulpitis, Risk factors

## Abstract

This retrospective matched case-control study aimed to identify potential risk factors associated with the development of dental abscesses during or after endodontic treatment. The study analyzed data from 133 patients (74 males, 58 females; mean age 43.1 ± 16.5 years) with abscess formation of endodontic origin and compared them with a 1:1 matched control group without abscess. Matching was performed based on age and the specifically treated tooth. Collected variables included preoperative symptoms, radiographic findings, type and stage of endodontic intervention, and systemic medical conditions. Statistical analysis used chi-square or Fisher exact tests to compare between groups. STROBE and PROBE guidelines were followed. Among 2637 maxillofacial infections of odontogenic origin, 526 cases (19.9%) were related to endodontic treatment. No statistically significant associations were found regarding age, sex, or underlying systemic diseases (*p* > 0.05). Teeth exhibiting apical radiolucency were at significantly higher risk for abscess development (*p* < 0.001). Teeth that had undergone definitive root canal filling were significantly less likely to develop abscesses compared to those left without temporary filling after trepanation (*p* < 0.001) or treated with intracanal medication and temporary filling (*p* = 0.009). The highest incidence of abscess formation was observed within the first four days post-treatment (*p* < 0.001). Within the limitations of this retrospective study, abscess formation during or after endodontic treatment was linked to local procedural and radiographic factors, whereas systemic diseases did not demonstrate a measurable influence. The absence of temporary sealing and the presence of apical radiolucency were identified as significant risk indicators and may warrant increased clinical attention, particularly in the early postoperative period. Given the retrospective design and limited sample size, these findings should be interpreted with caution and require confirmation in prospective studies.

## Introduction

In recent decades, effects of apical periodontitis and root canal treatments on systemic diseases have been investigated epidemiologically^1^. Oral infections and systemic diseases are closely linked and share many common risk factors^2–4^. Systemic diseases, particularly diabetes mellitus and cardiovascular disease, have been suggested to contribute to adverse endodontic outcomes, including impaired healing and reduced long-term tooth preservation^1,5–10^. The available evidence remains limited and inconclusive. However, although postoperative pain, flare-ups, and healing outcomes after endodontic treatment have been extensively studied, the risk factors associated with the development of submucosal abscesses requiring incision and drainage remain largely unknown. Apical radiolucencies may reflect persistent periapical infection and have been suggested as a potential risk factor for acute infectious exacerbations. Likewise, adequate temporary restorations are considered essential to prevent coronal leakage and reinfection of the root canal system between treatment appointments, although their influence on abscess formation has been insufficiently investigated. Identifying periods of increased risk for abscess formation could have important implications for patient care and follow-up strategies. Furthermore, evidence regarding the association between potential risk factors and the development of submucosal abscesses remains limited, and little is known about the timing of abscess formation during the various phases of endodontic treatment. An abscess is defined according to the National Cancer Institute, National Institutes of Health (Bethesda, MD, USA) as “An enclosed collection of pus in tissues, organs, or confined spaces in the body. An abscess is a sign of infection and is usually swollen and inflamed^11^”. However, a currently still valid guideline recommends, based on evidence, clinically differentiating between odontogenic infections in infiltrates and local odontogenic infections with or without a tendency to spread and with or without local or systemic complications^12^. Odontogenic infections arise because of bacterial inflammatory processes that originate in the teeth or periodontium^12^. They initially lead to inflammatory changes in the immediately adjacent tissue, but depending on the location, virulence of the pathogens, and individual immune competence, they can spread regionally or disseminate lymphogenically or hematogenically^12^. The clinical course can be mild, but—especially in cases of compromised immune status or unfavorable anatomical spread—it can also lead to severe or even life-threatening complications^12^. There is no uniform nomenclature to date; terms used in the literature include infiltrate, periodontal abscess, submucosal abscess, loge abscess, abscesses affecting multiple loges, abscess with a tendency to spread, odontogenic infection, and progressive abscess^12^.

The aim of this retrospective case-control study was to identify patient- and treatment-related factors associated with the development of submucosal abscesses during or after endodontic treatment, with particular attention to radiographic findings, temporary restorations, systemic diseases, and timing of abscess occurrence.

## Materials and methods

### Study design and data source

This retrospective matched case-control study aimed to compare patients in the case group, who developed abscesses during or following endodontic treatment, with a randomly selected control group of patients who underwent endodontic treatment without subsequent abscess formation. The study was approved by the Ethics Committee of the State Medical Association of Rhineland-Palatinate (application number: 2020-15180-retrospective; date: 30.07.2020). All patients included in the study provided written consent for the storage and use of their anonymized data for research purposes. The study follows the STROBE^13^ and PROBE^14^ guidelines. To minimize potential bias, a 1:1 matching procedure was used for this case-control design to select patients in the control group based on age, the specific endodontically treated tooth, and the endodontic treatment status (“treatment completed”/“treatment not completed”). Missing data were treated using multiple imputation, assuming that they were missing at random; all variables included in the analysis were entered into the imputation model and summarized across the imputed data sets. A sensitivity analysis was performed to assess the robustness of the results. Furthermore, attempts were made to avoid selection bias by only including patients who received both endodontic treatment and incision in the same university medical center (UMC). Data were extracted from the electronic records of the university medical center of the Johannes Gutenberg-University Mainz (Germany). The X-ray findings, the type of imaging, and whether periapical radiolucency was visible or whether there was a justifiable indication for an X-ray were intraoral images and/or panoramic images.

### Study sample and inclusion criteria

Between January 1, 2013, and December 31, 2017, a total of 2637 incisions procedures were performed at the dental clinics of the university medical center of the Johannes Gutenberg-University Mainz (Germany). Due to a lack of epidemiological data on the specific prevalence of submucosal abscess formation after endodontic treatment, the case number calculation was based on reported incidence rates of postoperative infectious flare-ups as described in large clinical cohort studies^15^. The calculation was performed with a statistical power of 80% at a two-sided significance level of α = 0.05. Within this cohort, 526 patients had previously undergone endodontic treatment, while 2111 had not. The inclusion criteria for this study required that patients had received prior endodontic treatment at the university medical center of the Johannes Gutenberg-University Mainz (Germany) and had undergone incision and drainage of an abscess as an outpatient procedure. Of the initial cohort, 393 patients were excluded because they were treated by their private dentist, leaving 133 cases that met the inclusion criteria for the study.

### Information extracted from patient records

- Demographic information (age at the time of the abscess, sex).
- Medical history (underlying systemic diseases).
- Treatment details (causative tooth, abscess location, symptoms, previous treatment, abscess management).

### Statistical analysis

The final dataset was analyzed using Microsoft Excel (Microsoft Corporation, Redmond, Washington, USA) and SPSS version 23 (IBM Corporation, Armonk, New York, USA). For quantitative variables, mean values and standard deviations were calculated. Qualitative data were reported as absolute numbers and relative frequencies. To explore the association between the outcome “abscess” and other variables, a binary logistic regression model was employed. Clinically relevant covariates available in the data set were included in the multivariable model. Variables such as surgeon experience, detailed quality parameters of canal preparation/filling, and socioeconomic factors were not consistently documented and therefore could not be considered. The tooth type was controlled by 1:1 matching. The Kruskal-Wallis test was used for comparing multiple independent groups, and where significant differences were identified, a post-hoc test was conducted using the Dunn-Bonferroni method. The Chi-square test was applied to assess the statistical significance of differences in nominal data frequencies, with Fisher’s exact test used when expexted frequencies were less than five. A significance level of *p* = 0.05 was set for all statistical tests.

## Results

**Fig. 1:**
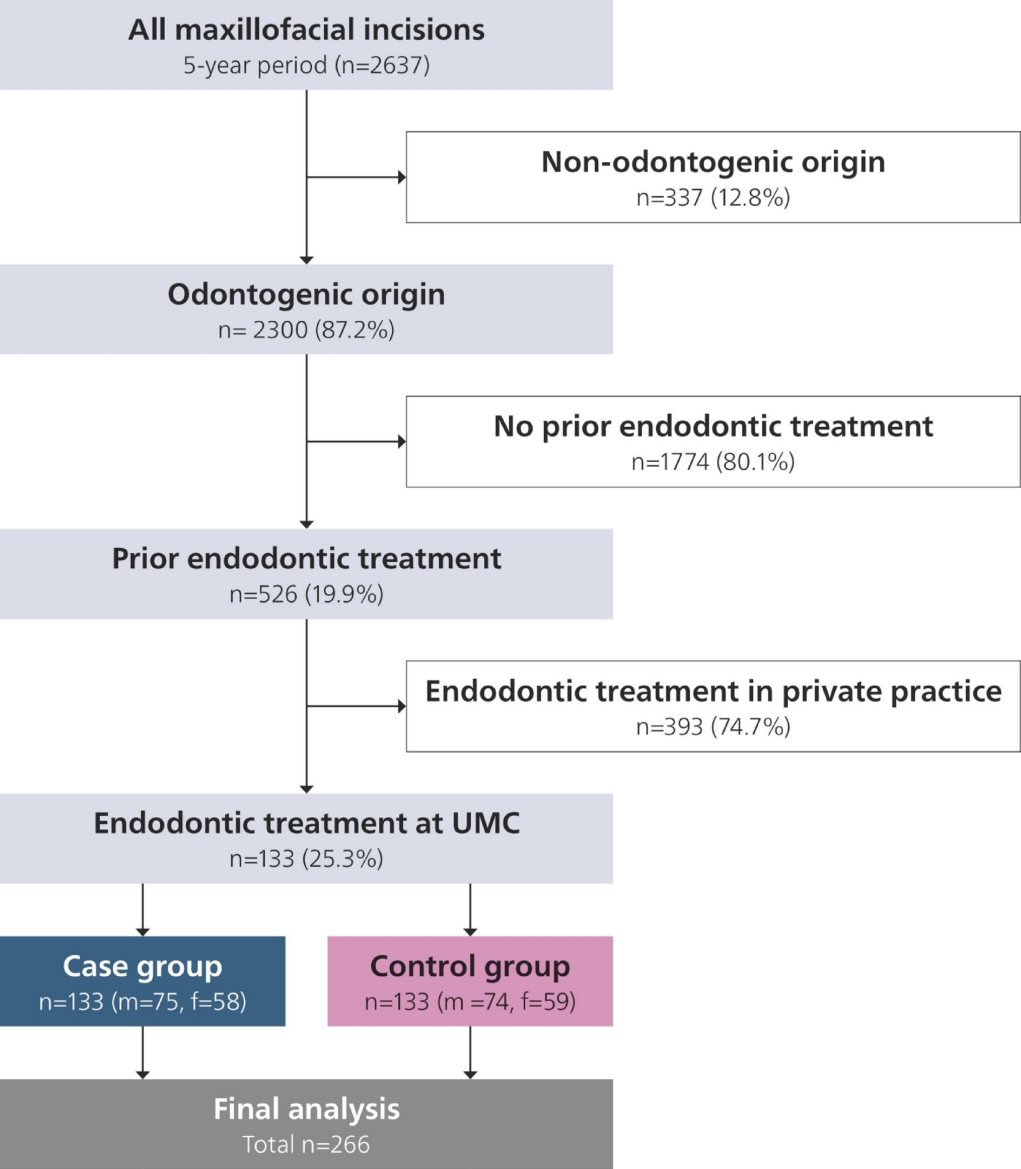
Flowchart.

Out of the 2637 incisions made in the maxillofacial region over the five-year period, 2300 (87.2%) were attributed to odontogenic infections. Among these, 526 cases (19.9%) involved teeth that had previously undergone endodontic treatment, with 133 patients (5.0%) having received this treatment at the university medical center of the Johannes Gutenberg-University Mainz (Germany). The sex distribution was comparable between the groups: the control group comprised 74 males (55.6%) and 59 females (44.4%), while the case group included of 75 males (56.4%) and 58 females (43.6%) (Fig. 1). The average age in the case group was 43.1 ± 16.5 years, with females averaging 45.9 years and males 40.9 years. A significant proportion (61.7%) of the case group was aged between 20 and 49 years at the time of abscess formation. The average age of the control group was matched to that of the case group, as age was one of the selected matching factors.

### Systemic diseases

**Fig. 2:**
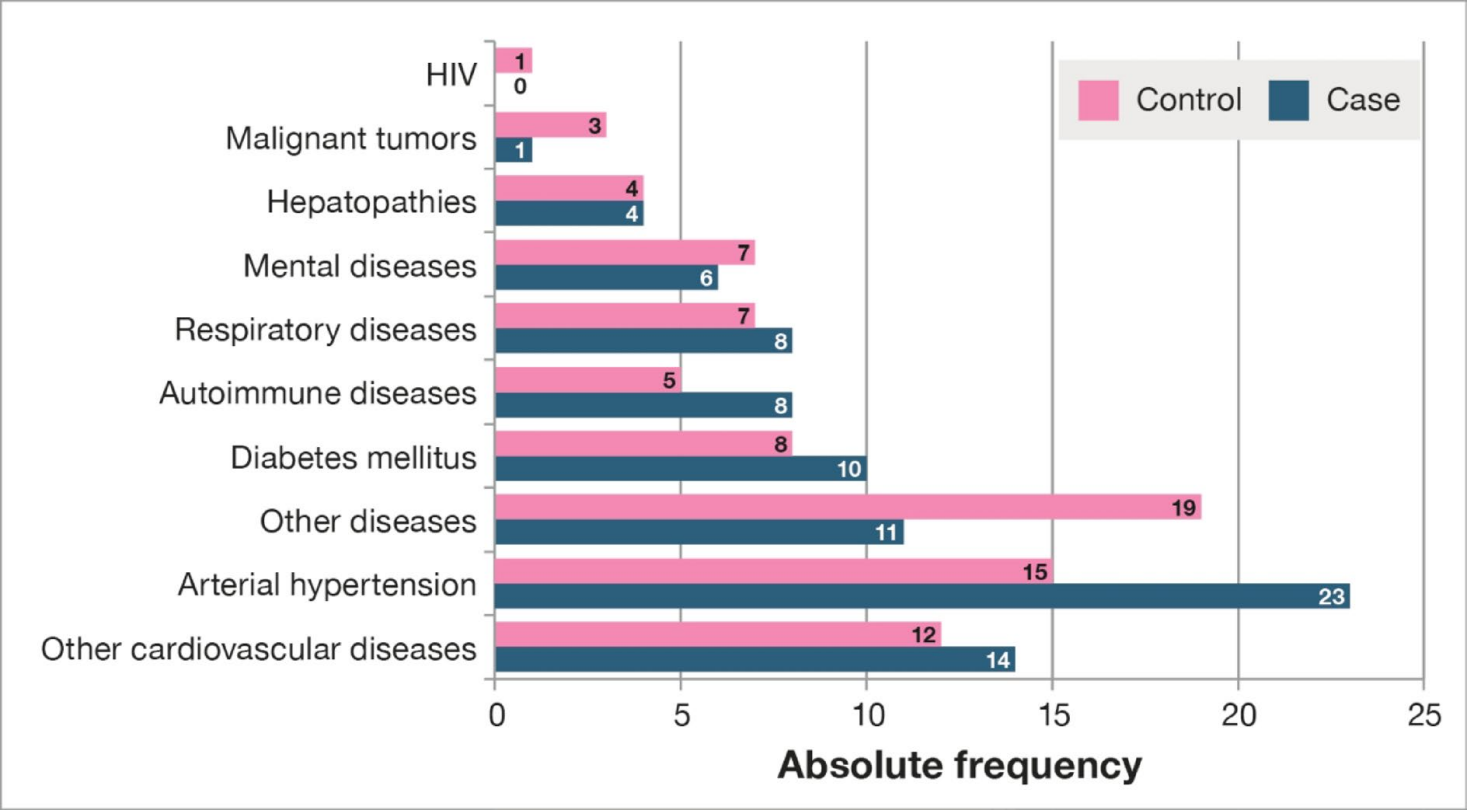
Systemic diseases.

**Fig. 3:**
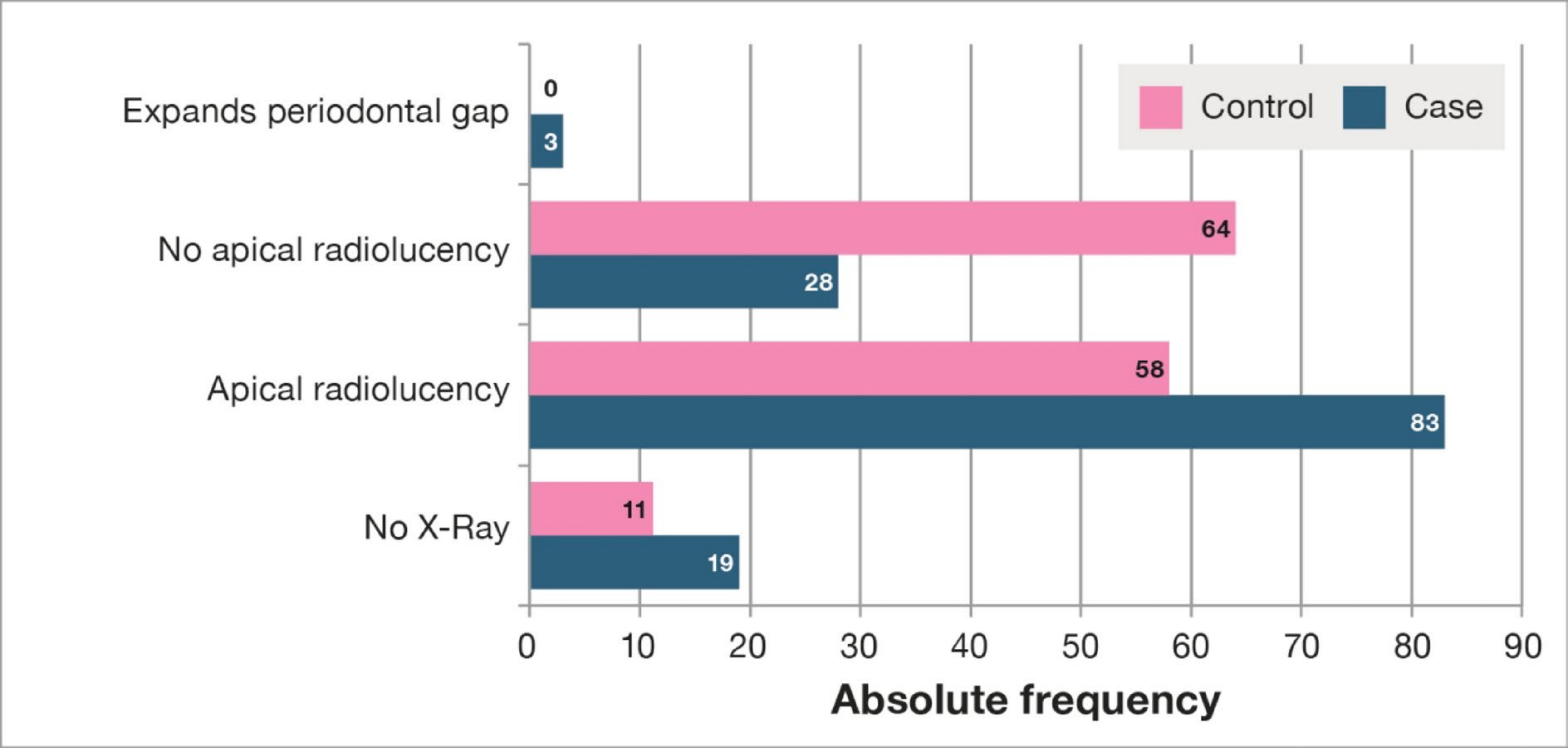
Radiological findings.

In the case group, 58 patients (43.6%) had one or more systemic diseases, 66 (49.6%) had no underlying conditions, and in 9 cases (6.8%), no medical history was recorded. A total of 86 conditions were documented due to some patients having multiple diseases. In the control group, 54 patients (40.6%) had one or more systemic diseases, 66 (49.6%) had no systemic conditions, and in 13 cases (9.8%), no medical history was available, resulting in a total of 81 documented conditions. Data on systemic diseases in both groups are illustrated in Fig. 2. The human immunodeficiency virus (HIV) infection listed in Fig. 2 was excluded from the binary logistic regression model as it was present in only one group. As shown in Table 1, all p-values from the binary logistic regression model exceeded the threshold of *p* = 0.05: Arterial hypertension (*p* = 0.191), other cardiovascular diseases (*p* = 0.744), respiratory diseases (*p* = 0.841), diabetes mellitus (*p* = 0.676), mental diseases (*p* = 0.729), hepatopathies (*p* = 0.962), autoimmune diseases (*p* = 0.425), and malignant tumors (*p* = 0.288). None of these factors reached statistical significance. In the case group, 83 patients (62.4%) showed apical radiolucency on the affected tooth, as depicted in Fig. 3. Apical radiolucency was statistically significantly more frequent in the case group (*p* = 0.003), while the absence of apical radiolucency was statistically significantly more common in the control group (*p* < 0.001).

**Table 1:** Binary logistic regression model (OR=Odds Ratio, CI = 95% confidence interval).

Systemic diseases	OR (exp^2^/β_1_)	CI of exp^2^/β_1_	*p*-value
Arterial hypertension	1.594	0.787–3.228	0.191
Other cardiovascular diseases	1.145	0.507–2.589	0.744
Respiratory diseases	1.113	0.391–3.172	0.841
Diabetes mellitus	1.228	0.468–3.225	0.676
Mental dieseases	0.821	0.268–2.517	0.729
Hepatopathies	0.967	0.236–3.956	0.962
Autoimmune diseases	1.586	0.504–4.993	0.425
Malignant tumors	0.317	0.033–3.092	0.288

### Relationship between endodontic treatment and time to abscess

Table 2 reveals two peaks in abscess formation timing. For teeth that were trepanned and left open without a temporary filling, 37 abscesses (62.7%) occurred between the first and fourth-day post-treatment. Abscesses in teeth with intracanal medication and temporary fillings were evenly distributed across all time periods. Among teeth that had been root canal-filled, most abscesses (*n* = 17, 63.0%) developed more than 30 days after treatment completion. The time differences between the case and control groups regarding abscess development were statistically significant (*p* < 0.001). A post-hoc test was conducted to identify significant differences between specific groups. Significant differences were found between the following pairs: (1) Trepanation without temporary filling vs. intracanal medication and temporary filling (*p* = 0.168), (2) Trepanation without temporary filling vs. root canal filling (*p* < 0.001), and (3) Intracanal medication and temporary filling vs. root canal filling (*p* = 0.009). A statistically significant increase in abscess formation was observed within the first four days following endodontic treatment compared to days 5 to 30 (*p* < 0.001). However, no significant difference was observed between days 1 to 4 and more than 30 days (*p* = 0.362), nor between days 5–30 and more than 30 days (*p* = 0.114).

**Table 2:** Relationship between endodontic treatment and time to abscess formation.

	Days until abscess formation	Absolute frequency (n)	Relative frequency (%)
Trepanation without temporary filling (n = 59)	1–4 days	37	62.7
	5–30 days	11	18.6
	> 30 days	11	18.6
Intracanal medication and temporary filling (n = 47)	1–4 days	18	38.3
	5–30 days	12	25.5
	> 30 days	17	36.2
Root canal filling (n = 27)	1–4 days	6	22.2
	5–30 days	4	14.8
	> 30 days	17	63.0

## Discussion

The objective of this study was to identify potential risk factors and the prevalence of submucosal abscess formation in relation to known systemic diseases, using a retrospective case-control design. Our findings corroborate previous research indicating that odontogenic sources are the predominant cause of maxillofacial infections^16^, with endodontically treated or retreated teeth accounting for nearly 20% of these cases^17^.

### Demographics and systemic diseases

No significant sex differences were observed in the occurrence of abscesses, consistent with earlier studies that also reported no significant gender disparities^16,18^. The mean age of patients in this study was 43.1 ± 16.5 years, aligning with other research where the affected population predominantly ranged from 34.9 to 49.5 years^19–21^. In our cohort, 61.7% of abscess cases occurred in patients aged 20–49 years, a distribution supported by Oglah et al.^22^, who found that 49.92% of root canal treatments were performed on individuals within this age group. While cardiovascular diseases, diabetes mellitus, and autoimmune disorders were slightly more prevalent in the case group, these differences were not statistically significant. This suggests that these systemic conditions do not significantly increase the risk of abscess formation following endodontic treatment, aligning with the findings of Furuholm et al.^23^ and Segura-Egea et al.^24^. Despite the biological plausibility—for example, due to a potentially impaired immune response or delayed tissue healing—no significant correlation could be demonstrated in the present cohort. This could be explained by the overall low incidence of abscess formation, the potentially adequate medication adjustment for systemic diseases, and standardized treatment protocols in the university setting. In addition, it can be assumed that abscess formation is predominantly influenced by local endodontic factors. Although diabetes mellitus is often cited as a risk factor for odontogenic infections^25,26^, our study did not confirm this association.

### Radiographic findings and abscess development

The results of this study are consistent with existing literature on the impact of apical radiolucency on the development of abscesses during or after endodontic treatment. Previous studies have established the role of apical radiolucency as a significant factor in abscess formation^27–29^. Research by Igbal et al.^15^ and Aksoy et al.^26^ highlights radiographically visible apical lesions as a key risk factor for endodontically induced abscesses, findings that are supported by our data and those of de Oliveira Alves^27^. The acute exacerbation of a previously asymptomatic chronic inflammation may occur when bacteria are introduced beyond the apical foramen during root canal preparation, disrupting the equilibrium between host defenses and pathogenic microorganisms, thereby triggering an inflammatory response.

### Impact of treatment type on abscess formation

Teeth that were temporarily sealed with intracanal medication exhibited a lower frequency of abscess formation between the first- and fourth-days post-treatment compared to those left open. This suggests that sealing the tooth with an endodontic temporary filling significantly reduces the risk of abscess development. Our findings are consistent with those of Udayakumar et al.^29^, who demonstrated that a temporary filling could prevent microorganism penetration into the root canal for up to seven days, although complete sterility cannot be guaranteed by any material. Teeth that had undergone root canal filling were the least likely to cause abscesses, with 63.0% of abscesses in this group occurring more than 30 days post-treatment. The possibility of recontamination, particularly within canal ramifications, cannot be ruled out. Both temporary and permanent root canal fillings are not completely impermeable to bacteria^30^. The instrumentation of the root canal can potentially introduce pathogenic microorganisms into the periapical area, thereby triggering an abscess^31^. However, based on the results of this study and supporting literature, ensuring a sufficient temporary seal after initiating endodontic treatment is crucial in reducing the risk of abscess formation. While temporary fillings can mitigate the risk, they cannot eliminate it. These findings highlight the importance of meticulous management and close monitoring of endodontically treated teeth to prevent postoperative complications.

### Limitations

This study has several strengths and weaknesses that should be mentioned. One strength of this study is the use of a well-defined, matched control group, which allows the results to be directly compared between patients with and without abscess formation after endodontic treatment. Another strength is the inclusion of multiple risk factors and the use of robust statistical methods for data analysis. The retrospective nature of the study, which increases the risk of information bias, and the lack of consideration of variables such as the treating dentist, equipment in the dental practice, technical variations in endodontic procedures such as the type and extent of root canal preparation, and bacterial contamination due to the corresponding cause, which could possibly influence the risk of abscesses, are among the weaknesses of this study. Furthermore, there is no agreed, standardized definition of the term “abscess” in the radiological context. While the abscess as a clinical-pathological entity is characterized by localized purulent inflammation, isolated radiological apical radiolucency does not allow a reliable distinction to be made between abscess, periapical granuloma, and radicular cyst. A definitive differentiation would only be possible histopathologically, which could not be performed in this retrospective study due to its nature. Generalization of the results to settings or populations other than the clinical setting and the general population may be limited due to the single-center nature of the study and the possibility that the different variables may affect the outcome.

Future research should focus on prospective studies of potential risk factors for the development of post-endodontic abscesses. The focus should be on the potential role of both systemic diseases and modern endodontic techniques and instruments. In addition, the influence of individual patient characteristics on treatment outcomes should be further investigated.

## Conclusions

Within the limitations of this study, the following conclusions can be drawn:

- The development of abscesses during or after endodontic treatment appears to be influenced by several risk factors, although systemic diseases, age, and sex do not seem to have a significant impact.
- The absence of an endodontic temporary filling may increase the risk of abscess formation.
- Teeth with apical radiolucency on radiographs are more prone to abscess development.

The highest risk period for abscess formation was within the first four days post- endodontic treatment, whereas most abscesses in root canal-filled teeth occured more than 60 days after treatment completion. These results suggest that particular attention should be paid to ensuring good temporary sealing and that teeth with apical radiolucency should be closely monitored. However, further studies are needed to confirm these correlations and to understand the possible causes in more detail.

## Data Availability

The datasets used and/or analysed during the current study available from the corresponding author on reasonable request.
